# Early Age of the First Myopic Spectacle Prescription, as an Indicator of Early Onset of Myopia, Is a Risk Factor for High Myopia in Adulthood

**DOI:** 10.1155/2021/6612116

**Published:** 2021-06-22

**Authors:** Wei-Yu Chiang, Yun-Wen Chen, Yu-Peng Liu, Yung-Hsun Liu, Pei-Chang Wu

**Affiliations:** ^1^Department of Ophthalmology, Kaohsiung Chang Gung Memorial Hospital and Chang Gung University College of Medicine, Kaohsiung, Taiwan; ^2^Graduate Institute of Clinical Medicine, College of Medicine, Kaohsiung Medical University, Kaohsiung, Taiwan

## Abstract

**Purpose:**

The present study investigated the risk factors for high myopia in adulthood, with a focus on the age at which children wore their first spectacles.

**Methods:**

Adults aged between 20 and 45 years were invited to complete a questionnaire about age, sex, current refractive error, high myopia in parents, early onset of myopia presented by the age of the first myopic spectacle prescription, refractive power of the first spectacles, and life habits at different educational stages. The associations between these factors and high myopia in adulthood were then evaluated and analyzed.

**Results:**

In total, 331 participants were enrolled. Their average refractive error was −4.03 diopters, and high myopia was noted in 27.5% of the study participants. Only 3.3% of participants had fathers with high myopia, while 6.0% had mothers with high myopia. The participants received their first myopic spectacle prescription at a mean age of 13.35 years, with a mean refractive error of −1.63 diopters. The significant risk factors for developing high myopia in adult life were earlier age of the first spectacles prescribed (*p* < 0.001), higher refractive power of the first spectacles (*p* < 0.001), mother with high myopia (*p*=0.015), and after-school class attendance in senior high school (*p*=0.018). Those who wore their first spectacles at <9 years of age were more predisposed to high myopia than those who did so at ≧13 years, with an odds ratio of 24.9.

**Conclusion:**

The present study shows that earlier onset of myopia, which is presented by the age of the first myopic spectacle prescription, higher myopic refraction of the first spectacles, mothers with high myopia, and after-school class attendance in senior high school are risk factors for high myopia in adulthood. It suggests that delaying the onset of myopia in children is important for the prevention of high myopia in later life.

## 1. Introduction

Myopia, also termed near-sightedness, is the most common ocular abnormality globally [1]. Myopia is not simply a refractive error; it is characterized by pathologic changes in axial length. Sight-threatening conditions of myopia are more commonly associated with high myopia than with mild to moderate myopia; they include cataracts, glaucoma, myopic choroidal neovascularization, foveoschisis, macular hole, chorioretinal atrophy, peripapillary deformation, choroidal/scleral thinning, rhegmatogenous retinal detachment due to peripheral retinal tears, and even blindness [2–5]. These sight-threatening complications can affect individuals of various ages and are more common in older adults. Mild to moderate myopia in childhood can ultimately lead to high myopia in adulthood, a concern that should be discussed with parents. Despite this, myopia was given little attention by the general population or eye care professionals in the past. Most people disregard it and think that myopia is just a minor inconvenience that can be managed by spectacles, contact lenses, or refractive surgical procedures [1]. Irreversible and highly prevalent myopia-related complications are already a severe public health burden [6].

The prevalence of myopia is particularly high in East Asia, especially in China, South Korea, Japan, Singapore, and Taiwan [7, 8]. For example, the annual incidence of myopia is 8–18% in 7- to 12-year-old Taiwanese children and only 2.2% in 12-year-old Australian children [9, 10]. Regardless of these racial and regional differences, both myopia and high myopia are projected to increase in prevalence globally [11, 12]. In 2000, 22.9% of the world population had myopia and 2.7% had high myopia. By 2050, the estimated equivalent figures are 49.8% and 9.8%, respectively [11].

Emmetropization is the process by which the length of the optical axis adjusts to its optical characteristics [13]. Myopization is an overshooting of emmetropization, with resultant axial elongation and thinning of the retina, choroid, and sclera, combined with further complications. The tissue that acts as the primary driver of this axial elongation has not been identified yet [13]. The identified risk factors for myopia include educational pressure, less time outdoors, prolonged near work, shorter reading distance, and a positive history of myopia in parents [14–19]. Several recent studies have aimed at developing preventive strategies based on the risk factors of myopia to inform future public health efforts [10, 20]. Myopia can be currently controlled by using atropine, orthokeratology, and multifocal contact lenses and partly controlled by increasing time outdoors [10, 20–22]. Because most cases of myopia develop in childhood, particularly during the school years, younger children, at the onset of myopia, tend to experience greater progression [13]. Thus, slowing the onset and progression of myopia is crucial to its control.

Questionnaires are noninvasive and convenient for collecting information about a specific population. Using information from a questionnaire, one study predicted refractive errors with reasonable sensitivity and specificity; as such, this method may assist future epidemiological studies of myopia screening [23]. Here, we used the questionnaire to determine the prevalence of myopia in middle-aged Taiwanese individuals and further identify the risk factors for high myopia.

## 2. Materials and Methods

### 2.1. Patients and Design

The present retrospective study used questionnaires to identify different activities and their correlation with the incidence of high myopia in the general population. Participants comprised individuals who presented for a general ocular health checkup or accompanied patients to the Ophthalmology Department of Kaohsiung Chang Gung Memorial Hospital in Taiwan, in 2014. None of the participants had any known ocular diseases other than refractive error, and none had undergone ocular surgery or myopia control. Individuals aged 20–45 years were selected based on the following criteria: stabilized refractive error [24], fewer presbyopia effects, and avoiding more recall mistakes than older individuals. This study adhered to the tenets of the Declaration of Helsinki and was approved by the Institutional Review Board of Kaohsiung Chang Gung Memorial Hospital.

**Figure 1:**
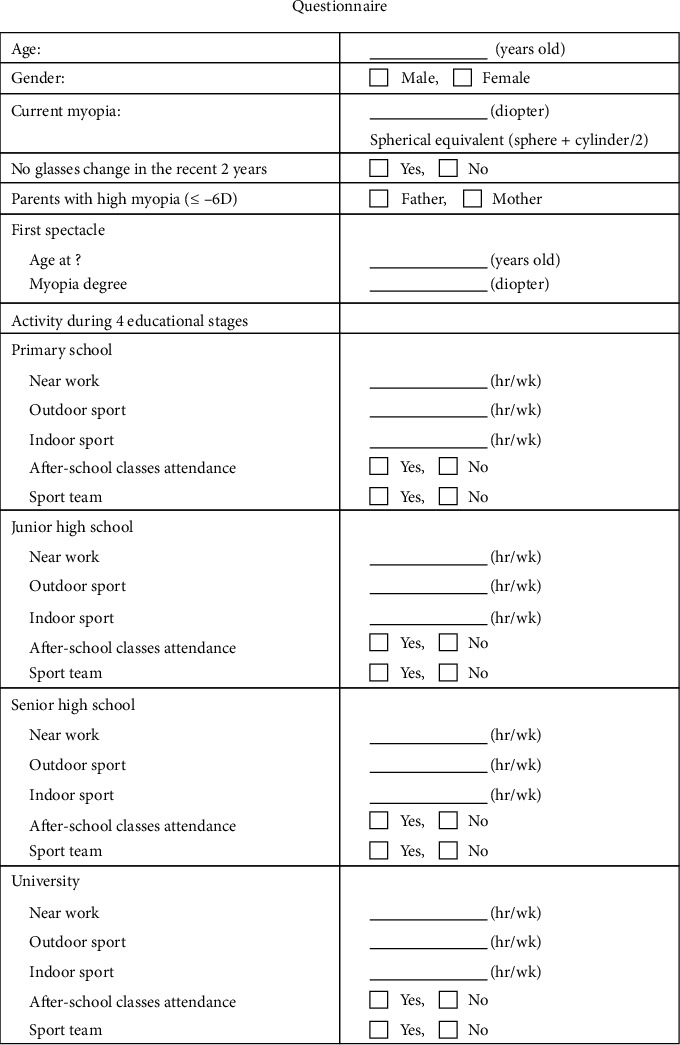
: A self-completed questionnaire was used to collect sex, age, current myopia degree, high myopia in parents, age at first spectacle use, refractive power of first spectacles, and life habits at four different educational stages.

A self-completed questionnaire (Figure 1) was used to collect the following clinical and demographic data: sex, age, current myopia degree, high myopia in parents, age at first spectacle use, refractive power of the first spectacles, and life habits at different educational stages (participation in after-school classes or sports, amount of time spent on work requiring eye focus (near work), and participation in indoor and outdoor sports in primary school, junior high school, senior high school, and university). Near work included the activities performed at short working distances, such as reading, writing, computer use, or watching TV [17]. The questionnaire was completed based on the recall and subjective information of the participants. This questionnaire demonstrated good test-retest reliability with an intraclass correlation coefficient (ICC) of 0.652–1.000. Participants who submitted incomplete questionnaires were excluded, as were those whose first spectacles had a positive refractive power for correcting hyperopia, those with their highest educational levels below university, and those who changed their glasses within the previous 2 years.

The right eye of each participant was selected from all participants. High myopia was defined as a spherical equivalent diopter with a refractive error of <−6.0 spherical equivalent diopters.

Figure 1 shows the questionnaire.

### 2.2. Statistical Analysis

The Kolmogorov–Smirnov test was used to test for normality as our data were not normally distributed. Continuous variables were expressed as median (interquartile range (IQR)). In the univariate analyses, the comparisons of patients with and without high myopia were performed using the Mann–Whitney U test for continuous variables and the chi-squared test for categorical factors. One-way repeated measure analysis of variance was used to analyze the time spent on (1) near work, (2) indoor sports, and (3) outdoor sports at different educational stages. The Greenhouse–Geisser adjustment was applied to the degrees of freedom. In the multivariate analyses, a stepwise logistic regression analysis was used to identify significant independent predictors of high myopia. Statistical significance was denoted by two-tailed *p* values of <0.05.

## 3. Results and Discussion

### 3.1. Demographic and Baseline Characteristics

A total of 331/520 (63.7%) of the participants completed the questionnaire (Table 1). Women constituted the majority of the participants (71.3%), and the overall average age was 32.2 years (range: 22–44 years). The mean age of male participants was 32.3 years, while that of the female participants was 33.1 years (*p*=0.137). The average refractive error was −4.03 diopters (range: −11.00 to 1.25 diopters); 27.5% of the participants had high myopia. A total of 3.3% of the fathers of the participants and 6.0% of their mothers had high myopia. The mean age at the prescription of the first spectacles was 13 years, and the mean refractive power at that time was −1.63 diopters. A total of 71.9% of the participants received their first spectacles before 15 years of age, while 35% received them before 12 years of age. Of those who received their first spectacles before 12 years of age, 58.6% developed high myopia during adulthood; in contrast, only 12.6% of those who received their first spectacles after 12 years of age developed high myopia (Table 2).

**Figure 2:**
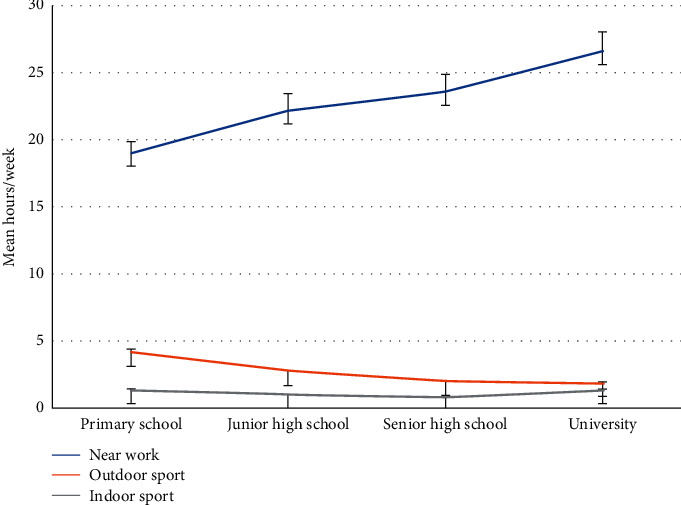
The mean of the time utilized in engaging in near work, outdoor sports, and indoor sports in four different educational stages. The time utilized in engaging in work that requires close eye concentration of near work showed a significant positive linear trend (*p* < 0.001), and outdoor sports showed a significant negative linear trend (*p* < 0.001). The time of indoor sports showed the lowest time at all four stages. Error bars indicate 1 standard deviation (SD).

Figure 2 shows data on the time spent on lifestyle habits at four different educational stages: (1) near work, (2) outdoor sports, and (3) indoor sports. The time spent on near work showed a significant linear increase from primary school to university (*p* < 0.001). In contrast, the time spent on outdoor sports showed a significant linear decrease (*p* < 0.001), while the time spent on indoor sports showed the lowest time at all four stages.

### 3.2. The Comparison between High Myopia and Non-High Myopia

Next, we compared the participants with high myopia and those with non-high myopia (Table 3). Age and sex were not significantly different between the groups. Although the proportion of fathers with high myopia was not significantly different, the number of mothers with high myopia was significantly higher in the high myopia group (12.1% vs. 3.8%; *p*=0.004). The age at first spectacle use was significantly younger in the high myopia group (11.05 years vs. 14.36 years; *p* < 0.001), and the high myopia group had a higher proportion of participants who used spectacles before 12 years of age (74.7% vs. 23.1%; *p* < 0.001). The refractive power of the first spectacles of the participants was also higher in the high myopia group (−2.28 vs. −1.38 diopters; *p* < 0.001). During the primary school period, the high myopia group spent more time on near work (21.21 vs. 18.22 hours/week, respectively; *p*=0.042); they also spent less time on indoor sports (0.54 vs. 1.60 hours/week; *p*=0.034) and outdoor sports (2.92 vs. 4.59 hours/week; *p*=0.033) during the same period. In junior high school, the high myopia group tended to spend more time on near work (24.23 vs. 21.45 hours/week; *p*=0.053). The high myopia group spent significantly more time on near work in both senior high school (26.45 vs. 22.52 hours/week; *p*=0.029) and university (29.73 vs. 25.42 hours/week; *p*=0.007). The frequency of after-school class attendance in senior high school was significantly higher in the high myopia group (54.9% vs. 41.7%; *p*=0.030).

### 3.3. Multivariate Analysis for Risk Factors of High Myopia

To identify the risk factors for high myopia, several significant factors (Table 3) were analyzed using logistic regression (Table 4). The multivariate analysis identified the risk factors for high myopia as the early age at first spectacle use (*p* < 0.001), high refractive power of the first spectacles (*p* < 0.001), mother with high myopia (*p*=0.015), and after-school class attendance in senior high school (*p*=0.018). The risk factors for high myopia in men were as follows: earlier age of first spectacle use (odds ratio (OR): 0.669; 95% confidence interval (CI): 0.532–0.840; *p*=0.001) and indoor sports time in university (OR: 0.660; 95% CI: 0.434–1.004; *p*=0.052). In contrast, women showed the following risk factors: early age at first spectacle use (OR: 0.604; 95% CI: 0.492–0.743; *p* < 0.001), high refractive power of the first spectacles (OR: 0.981; 95% CI: 0.975–0.988; *p* < 0.001), mother with high myopia (OR: 11.391; 95% CI: 1.732–74.903; *p*=0.011), and indoor sports time in university (OR: 1.408; 95% CI: 1.021–1.942; *p*=0.037).

### 3.4. The Effect of the First Spectacle Age on High Myopia in Adulthood

The age at first spectacle use significantly impacted high myopia prediction; thus, we analyzed the OR for high myopia based on the ages at first spectacle use (Table 5). Participants who were younger than 9 years or 10–12 years at the time of their first spectacle use had an OR of 24.9 and 5.3, respectively, compared with those who were older than 13 years. This result showed that a younger age at the time of first spectacle use was a predictor of more severe myopia progression in the future.

## 4. Discussion

This study showed that the factors including earlier onset of myopia represented as the first myopic spectacle prescription, higher myopic refraction of the first spectacles, having a mother with high myopia, and after-school class attendance in senior high school are risk factors for high myopia in adulthood. This is the first report that the reception of the first spectacle prescription at an earlier age was a biomarker of the early onset of myopia for predicting high myopia in later life. Therefore, the onset of school myopia should be delayed as much as possible by some interventions to reduce the prevalence of high myopia and its public health impact.

The prevalence of myopia is increasing globally and is particularly high in East Asian countries. A recent study showed that myopia prevalence was significantly and positively associated with higher age, female sex, parental myopia, and spending significant time indoors; in particular, playing with electronics carried the greatest risk [25]. Our study aimed to retrospectively review the possible risk factors of high myopia and found that an earlier onset of myopia was an important risk factor. The earlier onset of myopia in children is associated with a higher prevalence of myopia in adulthood.

Regarding parental myopia status, the rate of high myopia in the participants (27.5%) was much higher than that in their fathers with high myopia (3.3%) and that in their mothers with high myopia (6.0%), which is compatible with previous reports that the prevalence of both myopia and high myopia is increasing rapidly [11]. A family history of myopia has been reported as a risk factor for myopia [26–28]. Parssinen and Kauppinen indicated that women with myopic parents showed higher refractive power and faster/earlier myopia progression than those with nonmyopic parents [18]. It is often assumed that the impact of parental myopia is genetic. However, the difference in the attitudes of fathers and mothers toward education and behaviors toward visual care are significantly associated with myopia risk [29]. In this study, the difference in high myopia prevalence between the two generations suggests that genetics may not play a major role; however, environmental factors may be the major cause.

A recent meta-analysis showed that spending more time on near work was associated with a higher risk of myopia [17]. A higher educational level is associated with more myopic refraction [14, 30], which may be linked to the longer time spent on near work. This may partially explain the high prevalence of myopia in East Asia, where students spend much more time on studying and in after-school classes because there is fierce competition for higher education. The univariate analyses of the present study showed that the high myopia group spent more time on near work at all four educational stages, while the multivariate analysis indicated that the high myopia group undertook more after-school classes in senior high school.

In the present study, outdoor sports time was significantly lower in the primary school period in the high myopia group (Table 3). Spending more time outdoors is protective against myopia [10, 31, 32]. This is an important factor in public health efforts. Studies have found that bright outdoor light increases dopamine release, which simulates receptor activity, slowing axial elongation and decreasing the myopic shift [33–35]. In Taiwan, after outdoor activities were implemented in the Taiwan Student Vision Care Program, the prevalence of reduced visual acuity decreased [36]. Wu et al. recently reported that activities undertaken in moderate outdoor light intensity, such as in hallways or under trees, also have some protective effects against myopia development [20]. In the present study, although this factor did not reach significance on multivariate analyses, both outdoor and indoor sports times were significantly lower in primary school in the high myopia group (Table 3).

The age at first spectacle use has been discussed in previous studies. In a UK-based study, almost half of the individuals with myopia wore glasses only after 17 years of age [37]. Iribarren et al. reported that participants who were older at their first spectacle use tended to develop lower refractive error, but this relationship was not significant [38]. Another study indicated that subjects who developed myopia after the age of 20 years had low myopia [39]. The present study revealed that the mean refractive error of the first prescription in Taiwan was −1.62 diopters, at a mean age of 13 years. This finding is consistent with the clinical guidelines for childhood vision care in Taiwan, which suggest that the first spectacles should be prescribed once children have myopia of approximately −1.50 diopters and require refractive assistance in life. One study showed that spectacle use in myopic children with an error greater than −0.75 diopters would significantly improve their vision for daily life [40]. Wearing myopic spectacles may be a warning sign representing early age for the onset of myopia in children, especially in an epidemic area. Table 4 shows that younger age at first spectacle use was a predictor of more severe myopia progression in the future, with a high OR.

Based on these results, another interesting issue is that we should postpone spectacle use or undercorrection for myopic children who need refractive assistance. First, it is important to note that an early age at first spectacle use is the result of early myopia onset. In this study, we used the age at wearing the first spectacles to estimate the age of myopia onset. Early age at first spectacle use indicated early myopia onset. Second, regarding the use of glasses to control myopia progression, this question remains controversial and under investigation. The manipulations of optic correction in spectacles, including undercorrection or full correction, have been investigated for myopia control, but several studies have shown various results ranging from decreasing to worsening myopia progression [41–45]. We suggest that proper myopia correction using spectacles for learning and daily life, along with myopia control treatment for preventing high myopia in later life, such as atropine, orthokeratology, and multifocal contact lenses combined with sufficient daily time outdoors, would be a better strategy for school myopia control.

There were some limitations to the current study. First, the questionnaire collected self-reported data; therefore, the memory recall of participants was a major limitation. Second, a possible selection bias was noted. In this regard, the participants were selected from among those who came or accompanied someone for a general ocular health checkup. The educational level of all participants was above the university level. In addition, there were more female participants (71.3%) than male participants (28.7%). There are three reasons for this sex disparity. First, females are more careful about their health; second, females are more willing to accompany their family or friends to the hospital; and third, females were more willing to accept our invitation and complete the questionnaire. Third, the participants' purposes of spectacle use may not be for myopia only; other issues, such as astigmatism, may have played a role. Fourth, the time spent in after-school classes and sports team attendance was not quantified. The results indicated that after-school class attendance only in senior high school, but not other educational periods, was associated with high myopia. Perhaps, the time spent in after-school classes was much higher in senior high school than in other periods because the stress of the university entry system was the highest in senior high school. Further prospective longitudinal studies with larger sample size and objective refraction examinations are warranted to precisely identify the risk factors for myopia onset and progression.

## 5. Conclusions

The findings of the present study indicate that the predictive factors for high myopia include earlier age at first spectacle use, higher initial refractive power of spectacles, high myopia in the mother, and after-school class attendance in senior high school. In brief, earlier age at first spectacle use should be considered a warning sign for the development of high myopia in later life; thus, early myopia control should be emphasized.

## Figures and Tables

**Table 1:** Demographics and baseline results of all participants.

Clinical parameters	Results
Total (*n*)	331
Sex (male, (%))	95 (28.7%)
Age (years)	32.86 ± 4.63 (22–44)
Myopia (diopter)	−4.04 ± 2.63 (−11.00∼1.25)
High myopia	91 (27.5%)
Parents' high myopia	
Father	11 (3.3%)
Mother	20 (6.0%)
First spectacles	
Age (years)	13.35 ± 3.80 (2∼30)
Before 15 years old	238 (71.9%)
Before 12 years old	116 (35.0%)
Diopter	−1.63 ± 0.99 (−5∼−0.5)
Primary school	
Near work (hr/wk)	19.04 ± 15.68 (0∼106)
Outdoor sport (hr/wk)	4.13 ± 7.31 (0∼50)
Indoor sport (hr/wk)	1.31 ± 3.49 (0∼25)
After-school class attendance	130 (39.3%)
Sport team	46 (13.9%)
Junior high school	
Near work (hr/wk)	22.22 ± 16.55 (0∼105)
Outdoor sport (hr/wk)	2.71 ± 6.21 (0∼72)
Indoor sport (hr/wk)	0.97 ± 3.28 (0∼48)
After-school class attendance	250 (75.5%)
Sport team	19 (5.7%)
Senior high school	
Near work (hr/wk)	23.60 ± 18.38 (0∼105)
Outdoor sport (hr/wk)	1.97 ± 4.34 (0∼50)
Indoor sport (hr/wk)	0.74 ± 2.18 (0∼22)
After-school class attendance	150 (45.3%)
Sport team	13 (3.9%)
University	
Near work (hr/wk)	26.60 ± 19.34 (0∼132)
Outdoor sport (hr/wk)	1.87 ± 3.96 (0∼35)
Indoor sport (hr/wk)	1.34 ± 6.74 (0∼105)
After-school class attendance	20 (6.0%)
Sport team	14 (4.2%)

**Table 2:** The first spectacle age and the high myopia prevalence in adulthood.

The first spectacle age	High myopia in adulthood	Odds ratio	95% CI	*p* value
≦12 years vs. >12 years	58.6% vs. 12.6%	9.855	5.560–17.467	^*∗*^<0.001
≦15 years vs. >15 years	35.7% vs. 8.5%	6.000	2.312–15.573	^*∗*^<0.001

CI: confidence interval. ^*∗*^*p* < 0.05, statistically significant.

**Table 3:** Comparison between high myopia and non-high myopia.

Clinical parameters	High myopia (*n* = 91)	Non-high myopia (*n* = 240)	*p* value
Age (years)	32.51 ± 4.21	33.00 ± 4.78	0.334
Sex (male)	29 (31.9%)	66 (27.5%)	0.433
Current myopia (diopters)	−7.42 ± 1.61	−2.85 ± 1.64	<0.001^*∗*^
Parents' high myopia			
Father	4 (4.4%)	7 (2.9%)	0.503
Mother	11 (12.1%)	9 (3.8%)	0.004^*∗*^
First glasses			
Age (years)	11.05 ± 2.60	14.36 ± 3.76	<0.001^*∗*^
Age before 12 years	68 (74.7%)	48 (23.1%)	<0.001^*∗*^
Age before 15 years	85 (94.4%)	153 (73.9%)	<0.001^*∗*^
Refractive power (diopters)	−2.28 ± 0.89	−1.38 ± 0.91	<0.001^*∗*^
Primary school			
Near work (hr/wk)	21.21 ± 16.16	18.22 ± 15.45	0.042^*∗*^
Indoor sports (hr/wk)	0.54 ± 1.22	1.60 ± 3.99	0.034^*∗*^
Outdoor sports (hr/wk)	2.92 ± 5.73	4.59 ± 7.78	0.033^*∗*^
After-school class attendance	37 (40.7%)	93 (38.8%)	0.751
Sport team	11 (12.1%)	35 (14.6%)	0.558
Junior high school			
Near work (hr/wk)	24.23 ± 15.82	21.45 ± 16.79	0.053
Indoor sports (hr/wk)	0.49 ± 1.08	1.15 ± 3.79	0.188
Outdoor sports (hr/wk)	2.45 ± 5.11	2.81 ± 6.59	0.314
After-school class attendance	74 (81.3%)	176 (73.3%)	0.131
Sport team	6 (6.6%)	13 (5.4%)	0.681
Senior high school			
Near work (hr/wk)	26.45 ± 18.01	22.52 ± 18.44	0.029^*∗*^
Indoor sports (hr/wk)	0.57 ± 1.47	0.81 ± 2.39	0.583
Outdoor sports (hr/wk)	1.89 ± 3.26	2.00 ± 4.69	0.767
After-school class attendance	50 (54.9%)	100 (41.7%)	0.030^*∗*^
Sport team	4 (4.4%)	9 (3.8%)	0.787
University			
Near work (hr/wk)	29.73 ± 17.07	25.42 ± 20.04	0.007^*∗*^
Indoor sports (hr/wk)	0.64 ± 1.40	1.61 ± 7.85	0.766
Outdoor sports (hr/wk)	1.85 ± 4.27	1.88 ± 3.84	0.901
After-school class attendance	6 (6.6%)	14 (5.8%)	0.796
Sport team	3 (3.3%)	11 (4.6%)	0.604

**Table 4:** Logistic regression analysis for high myopia.

	Odds ratio	95% CI	*p* value
First spectacle age	0.649	0.564–0.747	<0.001
First spectacle refractive power	0.986	0.982–0.991	<0.001
Mother with high myopia (yes vs. no)	5.591	1.401–22.316	0.015
After-school class attendance in senior high school	2.488	1.158–4.620	0.018

CI: confidence interval.

**Table 5:** Logistic regression analysis of the age of the first spectacles for high myopia.

	Odds ratio	95% CI	*p* value
Age of the first spectacles			
≦9 years vs. ≧13 years	24.891	8.681–71.365	<0.001
10–12 years vs. ≧13 years	5.294	2.581–10.858	<0.001

CI: confidence interval.

## Data Availability

The data used to support the findings of this study are available from the corresponding author upon request.
